# What proportion of prescription items dispensed in community pharmacies are eligible for the New Medicine Service?

**DOI:** 10.1186/1472-6963-14-115

**Published:** 2014-03-07

**Authors:** Katharine M Wells, Matthew J Boyd, Tracey Thornley, Helen F Boardman

**Affiliations:** 1 School of Pharmacy, University of Nottingham, East Drive, University Park, Nottingham, UK; 2 Boots UK, D90 Thane Road, Nottingham, UK

**Keywords:** New Medicine Service, England, Prescription items, Pharmacy

## Abstract

**Background:**

The payment structure for the New Medicine Service (NMS) in England is based on the assumption that 0.5% of prescription items dispensed in community pharmacies are eligible for the service. This assumption is based on a theoretical calculation. This study aimed to find out the actual proportion of prescription items eligible for the NMS dispensed in community pharmacies in order to compare this with the theoretical assumption. The study also aimed to investigate whether the proportion of prescription items eligible for the NMS is affected by pharmacies’ proximity to GP practices.

**Methods:**

The study collected data from eight pharmacies in Nottingham belonging to the same large chain of pharmacies. Pharmacies were grouped by distance from the nearest GP practice and sampled to reflect the distribution by distance of all pharmacies in Nottingham. Data on one thousand consecutive prescription items were collected from each pharmacy and the number of NMS eligible items recorded. All NHS prescriptions were included in the sample. Data were analysed and proportions calculated with 95% confidence intervals used to compare the study results against the theoretical figure of 0.5% of prescription items being eligible for the NMS.

**Results:**

A total of 8005 prescription items were collected (a minimum of 1000 items per pharmacy) of which 17 items were eligible to receive the service. The study found that 0.25% (95% confidence intervals: 0.14% to 0.36%) of prescription items were eligible for the NMS which differs significantly from the theoretical assumption of 0.5%. The opportunity rate for the service was lower, 0.21% (95% confidence intervals: 0.10% to 0.32%) of items, as some items eligible for the NMS did not translate into opportunities to offer the service. Of all the prescription items collected in the pharmacies, 28% were collected by patient representatives.

**Conclusions:**

The results of this study show that the proportion of items eligible for the NMS dispensed in community pharmacies is lower than the Department of Health assumption of 0.5%. This study did not find a significant difference in the rate of NMS opportunities between pharmacies located close to GP practices compared to those further away.

## Background

In October 2011 the New Medicine Service (NMS) was introduced into community pharmacies in England to support patients prescribed new medicines for specified long term conditions (hypertension, type 2 diabetes, asthma/chronic obstructive pulmonary disease and patients receiving anti-coagulant/antiplatelet agents). The service aims to improve adherence to medicines and reduce medicines wastage [1]. The service consists of three parts, patient engagement (classified as usual care), the intervention and the follow-up. The intervention and follow-up stages consist of a semi-structured interview between the patient and pharmacist, which can be held in the pharmacy or by telephone, to support patients in their medicine taking and to highlight any problems they may be experiencing with their new medicine. The pharmacist would aim to address any problems where possible or refer the patient to their prescriber if necessary [1]. A patient is eligible to receive the NMS if they are prescribed a new medicine indicated for one of the eligible conditions and appears on the list of eligible medicines in the service specification [1]. The NMS can only be conducted where the patient (or, in the case of children, patient’s parent) can provide written consent.

Research conducted in the UK and Finland has found that adequate remuneration is an important facilitator to service provision and where payment is seen as inadequate, it can inhibit the uptake of services [2-5]. The payment structure for the NMS has been identified as a potential barrier to implementation [6].

The NMS service was initially funded in the first year by creating a fund of up to £55 m for the first 12 months of the service, which included payment for training and implementation. This was based on the assumption that 0.5% of all prescription items dispensed in community pharmacies would be eligible for the service [7]. This figure was the result of analysis by the Department of Health and is based on a theoretical figure due to data available at that time. The service has now been operating for over a year, making it timely to investigate the actual opportunity rate for the NMS in community pharmacies.

Community pharmacists and superintendent pharmacists (a strategic role, taking ultimate responsibility for pharmacists employed by their organizations, and the services they provide) [8] have questioned the assumption, reporting that the proportion of eligible items seen in their pharmacies is less than 0.5% of the items dispensed and have given several potential reasons for this. They suggested that the location of a pharmacy would affect the numbers of eligible items dispensed, as patients prescribed a new medicine by their doctor are more likely to get the prescription dispensed at the nearest pharmacy, even where this is not the pharmacy they regularly use. Additionally they proposed that this could mean that pharmacies co-located with health centres would see more eligible items than pharmacies located further away from GP practices. They also suggested that pharmacies that dispensed a higher proportion of Misuse of Drugs Act (MDA) prescriptions (instalment prescriptions used in the treatment of substance misuse) would have a lower proportion of eligible items compared to those who did not. In addition they reported that some items which appear eligible may not translate into a NMS opportunity, for example, if consent cannot be gained because the item is dispensed as part of a care home service (unpublished data; Wells K, Boyd MJ, Thornley T, Boardman HF). The implication of this is that pharmacies may not be able to access the full amount of funding allocated to the NMS if the rate of opportunities to provide the NMS is less that the theoretical rate of 0.5%. In April 2012 the Pharmaceutical Services Negotiating Committee (PSNC) acknowledged that the theoretical assumption may not reflect the rate of NMS opportunities for all pharmacies and has stated that it will be reconsidered in the future when data regarding the actual rate of NMS opportunities are available [7].

This study’s primary aim was to investigate the actual proportion of items dispensed in community pharmacies that are eligible for the NMS in practice and to compare this to the theoretical proportion of 0.5%. In addition the proportion of items eligible for the NMS at pharmacies close to General Practitioner (GP) practices was compared with those further away to determine whether distance from a GP practice affects the proportion of NMS eligible items presented in pharmacies.

## Methods

This study was carried out in pharmacies in Nottingham belonging to a large chain to minimise inter-pharmacy variation. At least one thousand consecutive prescription items were sampled from each pharmacy (a total of 8005 items) as for most pharmacies this represents several days prescriptions and enables several pharmacies in different locations to be sampled to provide a broader picture. This provided a balance between collecting large numbers of prescription items and being more representative of an individual pharmacy, and collecting from a wide range of pharmacy location types to be more representative of the pharmacy sector. The sample size software nQuery Advisor version 6 was used to conduct a sample size calculation based on the primary outcome to estimate a proportion with 95% confidence intervals to a power of 90%. This showed that 7852 prescription items were needed in total to detect a 0.5% difference (a 0.0025% change in prescription items eligible for the NMS), allowing for clustering effects, therefore data from at least 7852 prescription items would be collected.

The research team were advised by University Research Governance and the local Primary Care Trust Research and Development leads that ethical approval was not required as this study was an audit - the researcher conducting the study was a part time employee of the pharmacy chain from which the data was collected and there was no intervention. The study protocol was reviewed by a senior academic at the University of Nottingham and approval gained from the pharmacy chain head office and relevant area managers.

The 17 pharmacies belonging to a large multiple in Nottingham were grouped into three distances from GP practices: less than 100 meters, 100-500 meters and over 500 meters and the three groups were sampled to reflect as closely as possible the distribution of all pharmacies in Nottingham. These distances were chosen in order to distinguish between pharmacies co-located or next to GP practices, and pharmacies further away from GP practices. The distances were calculated by entering the pharmacy postcode into the “Find GP Services” page of the NHS choices website (http://www.nhs.uk). Pharmacies were excluded from the study if they dispensed less than 1000 prescription items per week (so that data collected in each pharmacy would be sufficient), if the pharmacy’s staffing levels required more than one person to receive and hand out prescriptions, meaning that more than one researcher would be needed to collect the data, or if the pharmacy primarily catered to an atypical demographic and are therefore unrepresentative meaning that the results from the pharmacy would be unlikely to reflect the average demographics seen by pharmacies. One pharmacy was excluded from the study because they dispensed less than 1000 items per week. Two pharmacies were excluded from the study because they required more than one person to receive and hand out prescriptions. One pharmacy was excluded due to its atypical demographic. This pharmacy was located within a university health centre and mainly caters to young people who are unlikely to require medicines for hypertension, COPD, type 2 diabetes or need anti-platelet agents or anticoagulants as these are conditions mainly affecting older people. Therefore this pharmacy could be expected to have a lower opportunity rate for the NMS than other pharmacies. Eight pharmacies were sampled from the remaining 12 possible pharmacies reflect the distribution of pharmacies in Nottingham according to distance from GP surgery. After gaining approval from the head office of the large chain and area managers, pharmacies were contacted directly by a researcher to be invited to participate in the study.

In each pharmacy the data were collected by a researcher taking in and handing out prescriptions to patients. Prescriptions were included in the study if they were a National Health Service (NHS) prescription, regardless of who collected the prescription, what type of NHS prescription it was, or whether the prescription was dispensed as part of a care home service. Prescriptions were only excluded from the study if they were private non-NHS prescriptions. A prescription item was eligible to receive the NMS if it was newly prescribed for hypertension, type 2 diabetes, asthma/COPD or was an anti-platelet or anti-coagulant agent, and the medicine was included in the list of medicines eligible for the NMS as specified in the service specification [1]. A prescription item meeting these criteria for the NMS was recorded in the study as eligible to receive the service regardless of who collected the prescription or whether it was part of a care home service. Therefore the study recorded the number of prescription items dispensed that were eligible to receive the NMS as well as actual NMS opportunities.

For each NHS prescription the researcher recorded the number of items on the prescription, whether the patient or a representative collected the prescription, if the prescription was delivered, whether the prescription was a MDA form or part of a care home service. Where an item was eligible for the NMS, the therapeutic class it fell into was recorded along with whether or not the NMS was offered and whether it was declined (and a brief reason why if provided by the patient). This study also recorded instances where items which were eligible for the NMS did not translate into an opportunity for the pharmacy to provide the service, for example where the patient was a child unable to consent to the service, or the patient was a care home resident. Data relating to private prescriptions were not recorded as only NHS prescriptions are eligible for the NMS. The data were collected between January and May 2013.

The data collected were inputted into the statistical software SPSS and frequency counts with percentages determined. Proportions were calculated with 95% confidence intervals and the confidence intervals used to compare the study results against the estimate that 0.5% of prescription items are eligible for the NMS [9].

## Results

In total 8005 items were recorded in 8 pharmacies in Nottingham (a minimum of 1000 items in each pharmacy) and of these 6080 items (76%) were NHS prescription items that were not MDA items or for care home residents (Table 1). Of the 8005 items recorded, 1965 (25%) were delivered to the patient or care home, and the remaining 6040 (75%) were collected from the pharmacies. Of the prescription items collected in the pharmacies, 28% (n = 1720) were collected by patient representatives.

**Table 1:** The types of prescription items included in the data collection

**Types of NHS prescription**	**Number of items recorded (%)**
Care home service	1665 (21%)
MDA	260 (3%)
Other	6080 (76%)
Total	8005

In this study 20 prescription items, 0.25% (95% CIs 0.14%-0.36%), were eligible for the NMS. This differs significantly from the assumption that 0.5% of prescription items are eligible for the NMS (z = 14.33). There were 17 opportunities (0.21%, 95% CIs 0.10%-0.32%) to provide the NMS (Table 2) as not all the eligible items translated into opportunities to offer the NMS. Three items were prescribed for the treatment of asthma in children who could not consent to the service (Table 3). The NMS was offered to 16 of the 17 patients that represented opportunities to provide the service. The one opportunity where the service was not offered was where a patient’s representative collected the dispensed prescription. The service was declined by 2 of the 16 patients offered the NMS, both of whom had been prescribed an anti-coagulant. Both patients stated the reason for declining the service was that they were receiving a lot of support from other healthcare professionals and felt that the support offered by the NMS was not needed.

**Table 2:** The percentage of NMS opportunities and NMS eligible items by distance from nearest GP practice

**Distance of pharmacy from nearest GP practice**	**Number of items collected**	**Eligible items**			**NMS opportunities**		
		**Number**	**Percentage with 95% CIs**		**Number**	**Percentage with 95% CIs**	
<100 m	n = 2002	7	0.35	(0.09-0.61)	6	0.30	(0.06-0.54)
100-500 m	n = 5004	11	0.22	(0.09-0.35)	9	0.18	(0.05-0.31)
>500 m	n = 999	2	0.20	-	2	0.20	-
Total	n = 8005	20	0.25	(0.14-0.36)	17	0.23	(0.10-0.32)

**Table 3:** The number of NMS eligible items and opportunities to provide the service by disease state from 8005 prescription items dispensed

	**Asthma/COPD**	**Hypertension**	**Type II diabetes**	**Antiplatelet/Anticoagulant**
Number of NMS eligible items	9	5	1	5
Number of NMS opportunities	6	5	1	5

There was no significant difference between the proportion of NMS eligible items at pharmacies close to GP practices compared with those further away (difference = 0.13%, 95% CIs -0.14% to 0.42%).

## Discussion

This study found that 0.25% of prescription items dispensed in community pharmacies are eligible for the NMS which is significantly different from the Department of Health’s theoretical assumption that 0.5% of prescription items would be eligible. It is possible that in calculating the 0.5% estimate the effect of some factors affecting the number of eligible items, such as prescriptions for care home residents, were underestimated, possibly explaining the difference between the observed number of eligible prescription items and the theoretical estimate.

Pharmacists were able to earn up to £55 m in the first year of the service based on pharmacists performing the NMS for 0.5% of their prescription items each month. In order to be remunerated for the NMS conducted, pharmacies claim payment each month for completed NMS in the same way that payment is claimed for NHS prescriptions dispensed. The results from this study would suggest that pharmacists were not able to access the full potential funding as the number of opportunities to carry out the NMS is less than 0.5% of their prescription items. NMS funding is outside the total agreed funding for pharmacy contractors, and if it is not earned then contractors are no longer able to access it and is not guaranteed to be made available for other public health initiatives. In April 2012 the PSNC communicated that theoretical assumption may not reflect the rate of NMS opportunities for all pharmacies and has stated that it will be reconsidered in the future [7]. This study suggests that the actual rate of NMS opportunities is less that the theoretical rate which means that it would be possible to widen the scope of the NMS by including other conditions eligible for the service to increase the number of opportunities a pharmacist has to conduct the NMS and consequently the number of patient who could benefit, without exceeding the funding limit.

Studies examining the provision of other UK pharmacy services have found that adequate funding is important to the success of a service [2-4]. This is not unique to the UK; research conducted in Finland has also found that pharmacies must be adequately reimbursed for providing a service if the service is going to be successful long term [5]. This study suggests that the assumptions used to calculate the funding envelope for the NMS are flawed as the actually opportunity rate to provide the service is less than the theoretical rate that underpins the potential funding available. This highlights the importance of evidence based methodologies to calculate funding allocation and applies not only to service in the UK but pharmacy services worldwide.

The results of this study suggest that a pharmacy’s opportunity rate to provide the NMS is less than the number of eligible items dispensed. In this study the reason for this was that eligible items were prescribed to patients who were not able to take part in the service because their age prevented them from being able to consent. The service to patients with asthma and is likely to be affected by this more than the other groups as children are less likely to have hypertension, type II diabetes or require anti-platelet agents or anti-coagulants, than asthma [10-16].

Of the 17 opportunities to offer the NMS in this study, there was just one occasion where the NMS was not offered to the patient, suggesting that the pharmacists engaged with the service take most available opportunities to provide the service. This contrasts with the early implementation of Medicine Use Reviews (MURs), where a national evaluation found that pharmacies offering MURs provided just 13.7% of the maximum number of MURs that could have been claimed for, despite this service being available for patients with any long term therapy [13]. A study carried out before the implementation of the NMS suggested that pharmacist engagement and NMS uptake would be greater than it was for MURs because when MURs were introduced it was seen as a change in direction for pharmacy requiring a cultural shift, whereas the NMS was seen as a natural extension of the role of community pharmacists [6].

In this study there were 16 occasions when the NMS was offered to patients and 2 occasions where the patient declined the service. The stated reason for this was the same in both instances, that the patient felt that they were receiving enough support from other healthcare professionals. There is evidence to suggest that the reason given by patients in a pharmacy for declining a service may not be the sole or entire reason the patient did not want the service [14], however both patients had been prescribed anti-coagulant agents and were attending anti-coagulant clinics so it is possible that the declines in this study indicate that some patients taking anti-coagulants are content with the existing support provided by other healthcare professionals.

In this study the most common condition receiving the NMS was asthma/COPD, followed by hypertension and anti-platelet agents/anti-coagulants with type II diabetes being the least common condition. National data published by the Pharmaceutical Services Negotiating Committee (PSNC) show that the most common NMS condition receiving the service is hypertension (54.4%), followed by asthma and COPD (26.4%), then type II diabetes (11.3%) with anti-platelet agent/anti-coagulant being the least common new medicines receiving the NMS (7.9%) [15]. The likely reason for the difference between the study data and the national data is likely to be due to the small numbers of NMS recorded in this study. If the sample size had been greater it is likely that the proportions of conditions would reflect the national data. Another possible reason for the difference between the study data and national data is that all the pharmacies sampled were in the same geographical location (Nottingham) and the demographics could potentially be different to demographics nationally.

In this study 28% of prescription items were collected by patient representatives, or proxies. While the proportion of prescriptions collected by patient representatives nationally is unknown, it is widely reported that around a third of requests for health information and non-prescription medicines in pharmacies are made by proxies [16-18].

The results of this study did not find a statistical difference between the proportions of NMS eligible prescription items dispensed in pharmacies co-located with GP practices and pharmacies further away. However, the study was not powered to test this so it is possible that with a larger sample size a difference may be detected.

### Limitations

One limitation of this study is that just 8 pharmacies in Nottingham were sampled (out of a total of 97) so it is possible that the study pharmacies would not reflect pharmacies locally. Therefore this study endeavoured to reflect the distribution of all pharmacies in Nottingham when sampling pharmacies. It was not possible to exactly match the distribution of pharmacies in Nottingham however, as there were just 2 pharmacies available in the <100 m group that matched the inclusion criteria.

Another limitation of the study is that all the pharmacies sampled were located in Nottingham and belonged to the same large chain, so it is possible that the pharmacies did not represent community pharmacy nationally. In addition the study collected 1000 items per pharmacy over a maximum of 6 days so there is a possibility that the 1000 items collected from each pharmacy did not represent a typical week’s prescription items for that pharmacy. However the pharmacies were selected to include a range of types and locations across the Nottingham area and data collection was spread over five months in an attempt to minimise these effects.

In this study pharmacies were excluded if they dispensed less than 1000 items per week or if the pharmacy’s staffing levels required more than one member of staff to take in and hand out prescriptions as this could have introduced potential selection bias. These demographic exclusions may reduce full generalizability and is a limitation of the study.

The pharmacies in this study were sampled over 5 months which could be a limitation as number and type of prescription items can vary over time, meaning that the data collected from an individual pharmacy may not reflect its long term dispensing patterns. However, by sampling pharmacies over 5 months the effect of seasonal prescribing patterns on the whole sample was reduced.

## Conclusions

The results of this study show that the proportion of items eligible for the NMS dispensed in community pharmacies is lower than the Department of Health assumption of 0.5%. Therefore pharmacy was not going to reach the maximum potential income of up to £55million in the first year, making it possible to widen the conditions eligible for the service to potentially benefit more patients without exceeding the available funding. This study did not find a significant difference in the rate of NMS opportunities between pharmacies located close to GP practices compared to those further away.

## Abbreviations

COPD: Chronic obstructive pulmonary disease; GP: General Practitioner; MDA: Misuse of Drugs Act; MUR: Medicine Use Review; NMS: New Medicine Service; PSNC: Pharmaceutical Services Negotiating Committee.

## Competing interests

KMW and TT are employees of Boots UK.

## Authors’ contributions

KMW carried out the data collection, data analysis, and drafted the manuscript. HFB helped analyze the data. All authors participated in the study design and revision of the manuscript.

## Pre-publication history

The pre-publication history for this paper can be accessed here:

http://www.biomedcentral.com/1472-6963/14/115/prepub
